# High prevalence of MRSA and ESBL among asylum seekers in the Netherlands

**DOI:** 10.1371/journal.pone.0176481

**Published:** 2017-04-25

**Authors:** Sofanne J. Ravensbergen, Matthijs Berends, Ymkje Stienstra, Alewijn Ott

**Affiliations:** 1 Department of Internal Medicine/Infectious Diseases, University of Groningen, University Medical Centre Groningen, Groningen, the Netherlands; 2 Department of Medical Microbiology, Certe, Groningen, the Netherlands; 3 Department of Medical Microbiology, University of Groningen, University Medical Centre Groningen, Groningen, the Netherlands; Rockefeller University, UNITED STATES

## Abstract

Migration is one of the risk factors for the spread of multidrug-resistant organisms (MDRO). The increasing influx of migrants challenges local health care systems. To provide evidence for both hospital hygiene measure and empirical antibiotic therapy, we analysed all cultures performed in asylum seekers between January 1^st^ 2014 and December 31^st^ 2015 for methicillin resistant *Staphylococcus aureus* (MRSA) and for multidrug-resistant Enterobacteriaceae (MDRE). We compared these with cultures from the Dutch patient population with risk factors for carriage of MDRO. A total of 7181 patients were screened for MRSA. 7357 *S*. *aureus* were isolated in clinical cultures. Of 898 screened asylum seekers, almost 10% were MRSA positive. Of 118 asylum seekers with *S*. *aureus* in clinical cultures almost 19% were MRSA positive. The general patient population had a 1.3% rate of MRSA in *S*. *aureus* isolates. A higher rate of Panton-Valentine leukocidin (PVL) positive strains (RR: 2.4; 95% CI: 1.6–3.4) was found in asylum seekers compared to the general patient population. In 33475 patients one or more Enterobacteriaceae were obtained. More than 21% of the asylum seekers were carrier of MDRE, most of them producing extended spectrum beta-lactamases (20.3%). 5.1% of the general patient population was MDRE carrier. It can be concluded that asylum seekers present with higher rate of MDRO compared to the general patient population. These results justify continued screening of asylum seekers to anticipate multidrug-resistant organisms during hospital care of patients.

## Introduction

The Netherlands has a low prevalence of multidrug-resistant organisms (MDRO) compared to other countries in Europe. For example, the proportion of invasive methicillin resistant *Staphylococcus aureus* (MRSA) isolates in the Netherlands is less than 1% compared to more than 10% in surrounding countries like Belgium and Germany. Rates up to 37–56% are found in Greece, Portugal and Romania in 2014 [1].

To prevent transmission of MRSA, the Netherlands uses a “search and destroy” policy [2]. This policy includes screening of patients from high risk groups, strict isolation at admission of patients suspected to be colonized with MRSA until cultures are shown to be negative, but also eradication treatment of MRSA [3–5]. This strategy was proven to be cost-effective and results in less death due to a bacteraemia [6].

High risk groups include patients who were admitted to hospitals in foreign countries within the last two months. However, optimal screening policy for travellers and asylum seekers is unclear. International travel is considered as a risk factor for acquisition of MDROs like extended spectrum beta-lactamases (ESBL) or carbapenemase-producing Enterobacteriaceae (CPE) [7]. We reported a 31% carriage rate of multidrug-resistant microorganisms, with ESBL-expressing *Escherichia coli* being the most common in asylum seeker patients admitted in a tertiary care University hospital in The Netherlands [8]. However, only a limited number of asylum seekers were screened and these patients may not have been representative for the total asylum seekers’ population.

Asylum seekers arriving in The Netherlands originate mainly from Syria, Iraq, Afghanistan and Eritrea [9]. These countries are assumed to have a higher carriage rate of MDRO [10, 11]. Due to the sudden high influx of asylum seekers and their expected higher carriage rate of MDRO, Dutch hospitals adopted various screening policies for MDRO in asylum seekers. A national screening policy is not available yet. More knowledge on MDRO carriage is needed to support decision making in national policies on hospital hygiene measures and empirical antibiotic therapy.

In this article, we will describe the rate of MRSA and multidrug-resistant Enterobacteriaceae (MDRE) among asylum seeker patients compared to the general patient population, based upon clinical and screening samples. This information may help to provide the best treatment and screening strategy for asylum seekers.

## Methods

### The asylum procedure and medical care for asylum seekers

Due to the centralised system of asylum application that is operated by the Netherlands, almost all asylum seekers have to report their arrival at the national reception centre in the north eastern province of Groningen. A minority reports at the national airport Schiphol. In the national registration centre, asylum seekers are registered and screened for active pulmonary tuberculosis by an X-ray. If (acute) medical care is needed, the patient is treated by the general practitioner at the national registration centre. If more specialised care is needed, the patient is referred to one of the regional hospitals.

### The Certe laboratory

The Certe laboratory performs microbiological diagnostics for both general (primary) and specialised (hospital based) health care for a catchment population of about one million inhabitants in the north east of the Netherlands. Since asylum seekers start their asylum procedure at the central organ for accommodating asylum seekers (COA) in this part of the Netherlands, the majority of samples taken in clinical care from asylum seekers who recently arrived in the country are sent to the Certe laboratory. For both asylum seekers and the general patient population about two third of all diagnostic samples come from one of the nine hospitals in the region. The remainder comes from primary care practitioners. Therefore, samples ranging from first to tertiary care are included.

### Selection of participants

A retrospective analysis was performed at the Certe laboratory. The study period and study population were defined by all routine cultures processed between January 1^st^ 2014 and December 31^st^ 2015.

Asylum seekers (and their offspring) were identified by their address if they were living in one of the asylum centres (COAs) in the region. A few asylum seekers living outside the COA were identified because the culture request was done by the physician at the COA health centre. All other patients in the laboratory database were categorised as the “general patient population”.

Much care was taken to clear double entries of asylum seekers from the database. Duplication had occurred in around 3% of cases due to different spellings of names and (rarely) a wrong date of birth. In the general patient population, duplication is prevented by a citizen service number which is unique for all citizens in the Netherlands. It should be noted that the general patient population in this study includes immigrants, temporary residents from abroad and ex-asylum seekers after receiving their residence permit.

This study was evaluated by the ethics committee and was waived in accordance with Dutch legislation owing to its retrospective nature (University Medical Centre Groningen, METc number 2016/516). No written informed consent was obtained from patients for the use of retrospective data but patient information was anonymized and de-identified prior to analysis.

### Methicillin resistant Staphyloccus aureus

MRSA was detected both actively and passively. Screening of patients for MRSA carriage can be regarded as an active method of detection whereas passive detection occurs in *S*. *aureus* isolates from clinical cultures performed to diagnose possible infection.

Screening was not done routinely in all asylum seekers, but most hospitals in the region adopted a screening regimen starting from April 2014. MRSA screening included a nasal, throat, perineum and only in some occasions skin culture. Most asylum seekers were screened in case of (anticipated) admission to the hospital.

Additionally to screening, all *S*. *aureus* isolated from clinical cultures were included in the analyses to study the difference in MRSA prevalence between asylum seeker patients and the general patient population.

Culture swabs collected from patients were transported in clear Amies media and all processed within one day after collection. Clinical cultures were incubated on two to six non-selective media, depending upon possible infectious agents, always including staphylococci and enterobacteriaceae. For screening cultures we used selective media. In case of MRSA screening we incubated a blood agar (Mediaproducts BV) for growth control, a selective Chrom ID MRSA (bioMérieux) and a Mueller Hinton broth with NaCl 2.5% (Mediaproducts BV). The selective broth was subcultured on solid media after one night incubation. Growth of *S*. *aureus* was confirmed by Staphaurex Plus (Oxoid), coagulase-test and Martineau gene PCR.

Antibiotic susceptibility of *S*. *aureus* was tested with the Vitek 2 automated system (bioMérieux). Isolates were additionally screened for beta-lactam resistance using the cefoxitin disk diffusion test [12] and MRSA confirmation was completed by detecting the *MecA* or *MecC* gene by PCR.

Of MRSA isolates the presence of the Panton-Valentine leukocidin (PVL) gene was tested with PCR. The PVL cytotoxin is associated with increased virulence of *S*. *aureus*. It is particularly associated with skin and soft tissue infections [13]. Molecular typing of MRSA isolates was done using Multiple Loci Variable Number Tandem Repeat Analysis (MLVA) performed by the Netherlands National Institute for Public Health and the Environment, which functions as the Dutch national reference centre for MRSA. The MLST clonal complex can be derived from most MLVA types.

### Multidrug-resistant Enterobacteriaceae

Similarly as for MRSA, MDRE can be detected both actively and passively. All Enterobacteriaceae isolated from clinical cultures were selected. As for MRSA routine MDRE screening of asylum seekers, started only halfway the study period. MDRE screening was performed using rectal swabs, which were processed within one day after collection. A growth control on blood agar and three selective solid media, a McConkey with ciprofloxacin 0,5 mg/l and gentamicin 2 mg/l (Mediaproducts BV), a ChromID ESBL and a ChromID Carbapenemase agar (both bioMérieux) were incubated.

Three patterns of MDRE were distinguished: Extended Spectrum Beta-Lactamase (ESBL), Fluoroquinolone plus Aminoglycoside Resistant Enterobacteriaceae (QARE) and carbapenemase-producing Enterobacteriaceae (CPE). Suspicious colonies were identified on species level by using MALDI-TOF. Only after a correct and plausible identification, the antibiotic susceptibility of Enterobacteriaceae was tested with the Vitek 2 system.

The antibiotic susceptibility of Enterobacteriaceae was tested with the Vitek 2 system. Presence of ESBL was confirmed with cefotaxime-clavulanate, ceftazidime- clavulanate and cefepime-clavulanate E tests (bioMérieux) [14]. Possible CPE was confirmed by PCR (Check-Points, Check-MDR CT102).

### Statistical analysis

Data were collected and analysed using Microsoft Excel and SPSS (version 2.22). Differences in proportions were tested for significance by Pearson’s uncorrected chi-squared test or the Fisher’s exact test as appropriate. Relative risk ratios (95% CI) were calculated for the virulence factor PVL and the MDRO rate.

## Results

In total 1071 asylum seekers were included in the study of which 973 had MRSA screening cultures or *S*.*aureus* in one or more clinical cultures and 859 had MDRE screening cultures or at least one of the enterobacteriaceae in clinical cultures. Of these 1071 asylum seekers 545 had cultures submitted to the laboratory by a primary care worker and 627 had cultures done by the second line (hospital) care.

### Methicillin resistant Staphylococcus aureus

During the study period 898 asylum seekers were actively screened for MRSA with a total of 3,106 cultures. Of these patients 87 (9.7%) were found to carry MRSA. In these patients MRSA was most often detected in their throat cultures (53; 61%), followed by nasal cultures (50; 57%) and perineum cultures (43; 49%).

In the same period 133 clinical cultures of 118 asylum seekers were positive with *S*. *aureus* isolates. Of these patients 22 (18.6%) carried MRSA (Table 1). 30.3% of the clinical isolates was a pus sample. No MRSA was obtained from blood cultures.

**Table 1:** Results of MRSA screening and MRSA among *S*. *aureus* isolates cultured from clinical samples, during 2014–2015, at the Certe laboratory.

	Number of patients	Number with MRSA	% with MRSA
MRSA screening			
General patient population*	6283	177	2.8%
Asylum seekers	898	87	9.7%
*S*. *aureus* in clinical samples			
General patient population	7239	92	1.3%
Asylum seekers	118	22	18.6%
totals from screening and clinical samples**			
General patient population	12989	269	2.1%
Asylum seekers	973	100	10.3%

* screened at hospital admission because of increased risk of MRSA carriage or contact investigation

** 533 of the general patient population and 43 asylum seekers had both screening and clinical cultures (number of totals less than sum of screening and clinical samples).

Of the general patient population 66 individuals were excluded from analyses because they had been identified with MRSA before 2014. Screening for MRSA in the general patient population was mainly done in persons at increased risk of MRSA carriage or in case of a contact investigation. In brief, patients considered to have an increased risk are patients working with livestock and patients who have been admitted to a foreign hospital over the last 2 months. More detailed information on risk factors for which screening on MDRO is performed in the Netherlands can be found in the national guidelines [3].

By screening, 177 new MRSA carriers were found in patients from the general patient population. A lower number of MRSA was found in patients with infections; in the clinical cultures from the general patient population only 92 new patients with MRSA were found (Table 1).

Both screening and clinical cultures of asylum seekers were significantly more often MRSA positive than of the general patient population (p < 0.001).

Each unexpected finding of MRSA in a clinical culture of an admitted patient was followed by screening of all contacts of this patient (including both caretakers and patients). Hospital acquired MRSA was defined as MRSA found in case of contact tracing around an index patient and if both index and contact strains were similar. According to that definition 25 of the 269 (9%) MRSA positive general patient (or caretaker) population had a hospital acquired MRSA, compared to one (1%) of the MRSA positive asylum seekers’ population.

### MRSA genotyping (Table 2)

**Table 2:** Genetic characteristics of MRSA isolates of the general versus the asylum seeker patient population.

	General patient population		Asylum seekers		Total	
Number of new MRSA*	269		100		369	
Livestock associated MRSA	127 (47.2%)		0		127 (32.0%)	
PVL** positive MRSA	48 (17.8%)		42 (42.0%)		90 (24.4%)	
Most prevalent MLST complexes***	**Number (%)**	**PVL-positive (%)**	**Number (%)**	**PVL-positive (%)**	**Number (%)**	**PVL-positive (%)**
CC398	124 (46.1)	0 (0.0)	0 (0.0)	0 (0.0)	124 (33.6)	0 (0.0)
CC5	46 (17.1)	3 (6.5)	5 (5.0)	0 (0.0)	51 (13.8)	3 (5.9)
CC8	27 (10.0)	25 (92.6)	7 (7.0)	0 (0.0)	34 (9.2)	25 (73.5)
CC22	12 (4.5)	0 (0.0)	20 (20.0)	0 (0.0)	32 (8.6)	0 (0.0)
CC30	11 (4.1)	10 (90.1)	3 (3.0)	2 (66.7)	14 (3.8)	12 (85.7)
CC45	11 (4.1)	0 (0.0)	0 (0.0)	0 (0.0)	11 (2.9)	0 (0.0)
CC1	2 (0.7)	1 (50.0)	27 (27.0)	18 (66.7)	29 (7.9)	19 (65.5)
CC88	2 (0.7)	2 (100.0)	15 (15.0)	9 (60.0)	17 (4.6)	11 (64.7)
CC80	6 (2.2)	6 (100.0)	5 (5.0)	5 (100.0)	11 (3.0)	11 (100)

* MRSA: Methicillin resistant *Staphylococcus aureus*

** PVL: Pantone-Valentine leukocidin

*** MLST: Multilocus sequence typing

MRSA strains of asylum seekers were significantly more often PVL positive (42.0%) than of the general patient population (17.8% (RR: 2.4; 95% CI:1.6–3.4)). A high proportion (47.2%) of MRSA in the general patient population was livestock associated: CC398 or MLVA complex MC2236. This type of MRSA was never PVL positive. None of the asylum seekers had a livestock associated MRSA. After excluding livestock associated MRSA, 33.8% of the remaining general population patients’ MRSA was PVL positive, still lower than the proportion of PVL in asylum seekers’ MRSA, but this difference is not statistically significant (*p* = 0.19).

CC398 was by far the most common type of MRSA among the general patient population. In asylum seekers MRSA CC1 was the most prevalent type and 18 (66.7%) of these strains were PVL positive. CC5, CC8 and CC22 were isolated in both patient categories, but CC22 significantly more often in asylum seekers (*p* < 0.001). CC8 was evenly distributed in both groups, but remarkably none of the asylum seeker strains was PVL positive, whereas 25 (92.6%) of the general patient population strains were PVL positive. MLVA complex MC0281 (related to CC88) was often found among asylum seekers. Of these strains 9 (60%) were PVL positive.

### Multidrug-resistant Enterobacteriaceae

For this part of the study we included 58,748 Enterobacteriaceae isolated from clinical cultures. Of these, 215 isolates were obtained from asylum seekers and 58,533 from the general patient population. Among asylum seekers 38 (17.7%) of their isolates were MDRE versus 2,554 (4.3%) of isolates from general population patients. 78.1% of the clinical isolates was obtained from urine samples.

Screening was mostly done in patients who had previously been colonized with MDRE or after admission in a foreign hospital. Differently than for MRSA, no contact tracing was done in case of MDRE. In total, sensitivity testing was performed on 5,300 isolates from screening cultures. Among those 1,557 (29.4%) were MDRE.

Details of MDRE carriage detected by screening or in clinical cultures are given in Table 3. Most MDRE were of the ESBL type. Many strains carried more than one resistance pattern.

**Table 3:** Number of patients tested and proportions of multidrug-resistant Enterobacteriaceae (MDRE) in screening and clinical cultures, during 2014–2015, at the Certe laboratory.

	Number of patients	% MDRE	% ESBL	% QARE	% CPE
MDRE screening					
General patient population*	1763	24.4%	16.3%	12.5%	0.06%
Asylum seekers	751	21.0%	20.0%	4.4%	0.1%
Relative Risk (95% CI)		0.9 (0.7–1.0)	1.2 (1.0–1.5)	0.4 (0.2–0.5)	2.4 (0.1–85.6)
Enterobacteriaceae in clinical samples					
General patient population	31,798	4.6%	2.7%	2.6%	0.02%
Asylum seekers	150	21.3%	19.3%	7.3%	0.0%
Relative Risk (95% CI)		4.6 (3.3–6.3)	7.2 (5.0–10.0)	2.8 (1.5–5.1)	0.0 (0–196.5)
totals from screening and clinical samples**					
General patient population	32,616	5.1%	3.2%	2.8%	0.02%
Asylum seekers	859	21.4%	20.3%	4.9%	0.1%
Relative Risk (95% CI)		4.2 (3.7–4.8)	6.3 (5.5–7.3)	1.75 (1.3–2.4)	6.33 (0.3–52.4))

* screened at hospital admission because of increased risk of MDRE carriage

** 945 of the general patient population and 42 asylum seekers had both screening and clinical cultures (number of totals less than sum of screening and clinical samples)

CPE were rarely found. Among asylum seekers no clinical culture contained CPE and only one patient had a CPE *E*. *coli* in his screening culture.

Table 3 shows a striking difference in MDRE rates in asylum seekers compared to the general patient population (21.4% vs. 5.1%). This difference mainly results from the clinical samples. A relatively small number of the general patient population was screened for MDRE because of specific risk factors. In these patients the carriage rate of MDRE is similar to the rate in asylum seekers, except for carriage of the QARE type resistance, which was less common in asylum seekers (*p* < 0.001).

In clinical cultures MDRE, ESBL and QARE were all significantly more prevalent among asylum seekers than general population patients (*p* < 0.001).

## Discussion

The aim of this retrospective study was to assess carriage rates of MRSA and multidrug-resistant Enterobacteriaceae (MDRE) among asylum seekers and to compare these to the general patient population. The data we used is unique because of the large number of patients from primary till tertiary care and included both screening and clinical cultures. For this reason, the results can be expected to represent the overall asylum seekers’ population arriving in the Netherlands during the study period. Knowledge on MDRO carriage is needed to provide evidence for both hospital hygiene measures and empirical antibiotic therapy.

Particularly higher carriage rates of MRSA were found in the asylum seekers’ population than in the Dutch patient population with risk factors for MDRO carriage. Among asylum seekers less MRSA was found by screening than in clinical cultures, suggesting that these MRSA are either more pathogenic or these infections were not responding to treatment and therefore more likely to be cultured than infections caused by beta-lactam sensitive *S*. *aureus*. The higher pathogenicity of the MRSA is supported by PVL rates which were twice as high in MRSA from clinical cultures as in strains obtained by screening. The MRSA prevalence of the general population may be better reflected by clinical cultures than screening cultures, because the latter was only done in a small subset of either high risk or contact patients.

Higher MRSA and MDRE carriage rates in asylum seekers were described before [15,16] as was the presence of PVL in asylum seeker strains [17,18]. Reinheimer et al [15] detected a 61% MDRO rate in refugee patients admitted to a University Hospital. This is much higher than the rate found in our study and could be explained by a difference in study population. This tertiary care patient category may have higher MDRO rates than those requiring first or second line care. Samples from different primary health centres and hospitals reflect both first, second and tertiary care. This increases the external validity of our study results since it is expected to reflect asylum seekers with different health care needs.

Based on epidemiological data, hospital acquired MRSA was more common in the general patient population than in the asylum seeker population. The lack of data on medical history including hospital admissions could have led to an underestimation of hospital acquired MRSA in the asylum seekers.

High MRSA rates in the asylum seekers’ population may be due to a high prevalence in their homelands and thus reflect long-term stable carriage, or they can be due to transmission between asylum seekers. The finding that among 104 asylum strains 56 different MLVA types were found, argues for the first hypothesis. Only two MLVA types were isolated more than ten times. One of these types, MT0281, belonging to MC0281 (CC88) was found in 12 strains, half of which were PVL positive. MT4594, belonging to MC0001 (CC1), was found 11 times of which all strains were PVL positive. More information on the MC-types in the country of origin is needed to interpret the variety of MC-types carried by asylum seekers.

The asylum seekers’ population carriage rates of MDRE were higher than the carriage rate in the general patient population. As only a small number of high risk general population was screened for MDRE, the most valid comparison between the two study groups can be done with MDRE rates found in clinical cultures. Asylum seekers have 4 to 5 time higher rates of MDRE than the general population and particularly more ESBL. International travel is described as a risk factor for the colonization of multidrug-resistant organisms in travellers. [19,20] and a higher carriage rate of ESBL was also described in refugee minors arriving in Germany. However, this study group is only represented by refugee minors under 18 years old and may not represent the overall population [21]. Interestingly, differences in QARE rates are only small in our study. CPE was rare in both groups, but monitoring is necessary since the carriage rate of CPE may vary if the population of asylum seekers changes and includes e.g. more patients with chronic diseases and earlier hospitalizations.

Reasons for higher MDRE carriage in asylum seekers may be similar as for MRSA carriage. As MDRE is more diverse and we know less from typing of these organisms we cannot speculate about the origin of these organisms.

The method used for MDRO screening is sensitive and efficient. However, a limitation of this method is that MDRO may be missed if they colonized a different body site than the regular ones. Another limitation is that MDRO found by screening may only represent temporary carriage and may have little or none clinical relevance. Still people with MDRO are registered and health-care workers are alerted whenever they take care of an MDRO carrier.

The total carriage rate of MRSA in the Dutch patient population with risk factors exceeds the prevalence observed in the overall Dutch population (<1%). The screening was performed in a small subset with risk factors of MRSA carriage like working with livestock or close contact with known MRSA carrier. Similarly the patients selected for MDRE screening had risk factors like foreign hospital admissions, earlier positive screening results. The prevalence of the general patient population may be better reflected by clinical cultures than screening cultures.

In asylum seekers there was no difference between MDRE in screening and clinical cultures, suggesting that both are a good reflection of MDRE rates in the asylum seeker patient population. This provides evidence for screening for MDRE in asylum seekers arriving in the Netherlands. Carriage of ESBL may be a threat especially for children since treatment options for children are limited [22]. The current study is performed in the Netherlands where ESBL carriage is still considered a reason to screen and isolate patients. The study group represents the Dutch asylum seekers’ population but consists of a heterogeneous group of people originating from many countries with different travel routes and possible hospital admissions before arriving in the Netherlands. To customize the Dutch screening policy additional information on these risk factors is needed.

Compared to asylum seekers the general patient population had more clinical than screening cultures, particularly with Enterobacteriaceae. Only a minority of the Dutch patient population with risk factors is screened for MDRE, namely those at high risk of resistance. That explains the much higher yield of MDRE from screening than clinical cultures.

In conclusion, our study shows significantly higher rates of MRSA and MDRE among the asylum seekers’ population than the general patient population. These differences justify screening of the asylum seekers’ population at admission in the hospital as these organisms may be a threat to the patient and transmission in the hospital should be prevented.
